# Inclusion Complex of Zerumbone with Hydroxypropyl-****β****-Cyclodextrin Induces Apoptosis in Liver Hepatocellular HepG2 Cells via Caspase 8/BID Cleavage Switch and Modulating Bcl2/Bax Ratio

**DOI:** 10.1155/2013/810632

**Published:** 2013-05-08

**Authors:** Nabilah Muhammad Nadzri, Ahmad Bustamam Abdul, Mohd Aspollah Sukari, Siddig Ibrahim Abdelwahab, Eltayeb E. M. Eid, Syam Mohan, Behnam Kamalidehghan, Theebaa Anasamy, Kuan Beng Ng, Suvitha Syam, Ismail Adam Arbab, Heshu Sulaiman Rahman, Hapipah Mohd Ali

**Affiliations:** ^1^UPM-MAKNA Cancer Research Laboratory, Institute of Bioscience, University Putra Malaysia (UPM), Serdang, 43400 Selangor, Malaysia; ^2^Department of Chemistry, Faculty of Science, University Putra Malaysia (UPM), Serdang, 43400 Selangor, Malaysia; ^3^Department of Pharmacy, Faculty of Medicine Building, University of Malaya, 50603 Kuala Lumpur, Malaysia; ^4^Medical Research Center, Faculty of Medicine, Jazan University, P.O. Box 114, Jazan 45142, Saudi Arabia; ^5^College of Pharmacy, Qassim University, P.O. Box 2055, Buraydah 6800, Saudi Arabia; ^6^Department of Microbiology and Pathology, Faculty of Veterinary Medicine, University Putra Malaysia (UPM), Serdang, 43400 Selangor, Malaysia; ^7^Department of Chemistry, Faculty of Science, University of Malaya, 50603 Kuala Lumpur, Malaysia

## Abstract

Zerumbone (ZER) isolated from *Zingiber zerumbet* was previously encapsulated with hydroxypropyl-**β**-cyclodextrin (HP**β**CD) to enhance ZER's solubility in water, thus making it highly tolerable in the human body. The anticancer effects of this new ZER-HP**β**CD inclusion complex via apoptosis cell death were assessed in this study for the first time in liver hepatocellular cells, HepG2. Apoptosis was ascertained by morphological study, nuclear stain, and sub-G1 cell population accumulation with G2/M arrest. Further investigations showed the release of cytochrome c and loss of mitochondrial membrane potential, proving mitochondrial dysfunction upon the ZER-HP**β**CD treatment as well as modulating proapoptotic and anti-apototic Bcl-2 family members. A significant increase in caspase 3/7, caspase 9, and caspase 8 was detected with the depletion of BID cleaved by caspase 8. Collectively, these results prove that a highly soluble inclusion complex of ZER-HP**β**CD could be a promising anticancer agent for the treatment of hepatocellular carcinoma in humans.

## 1. Introduction

The use of plants as the preferential treatment for cancer has been known for centuries. To date, about 3000 plant species have been identified as possessing anticancer properties. This piece of information is later utilized and investigated by scientists in search for better compounds to act as anticancer agent [1]. One of the most important benefits of using these plants for health purposes is the availability of anticancer agents useful for treatments [2]. This is proven by the fact that more than 60 types of clinical chemotherapy drugs used as anticancer agents are plant derived. Amongst this, a number of new, promising anticancer agents in clinical drug development based on selective activity against cancer-related molecular targets include flavopiridol and combretastin-A4-phosphate, while other anticancer agents which failed in earlier clinical studies have stimulated renewed interest [1].

*Zingiber zerumbet* is a member of the family Zingiberaceae. Other plants that belong to this family include *Zingiber cassumunar *and* Zingiber officinale* [3]. The plant is also known as lempoyang in Malaysia, Ghatian and Yaiimu in India, and Jangli adha in Bangladesh [4–6]. It can be found in the moist forest, beach thickets and mangrove margin. *Z. Zerumbet *extracts have been used traditionally for treating fever, headaches, asthma, indigestion, diarrhea, severe sprains, inflammation, constipation, and toothache, while the Malays use the rhizomes extract to cure edema and worm infestation in children [7, 8]. It was found that the oils obtained from the rhizomes were rich in zerumbone (37%), *α*-humulene (14.4%), and camphene (13.8%) [9].

**Figure 1:**
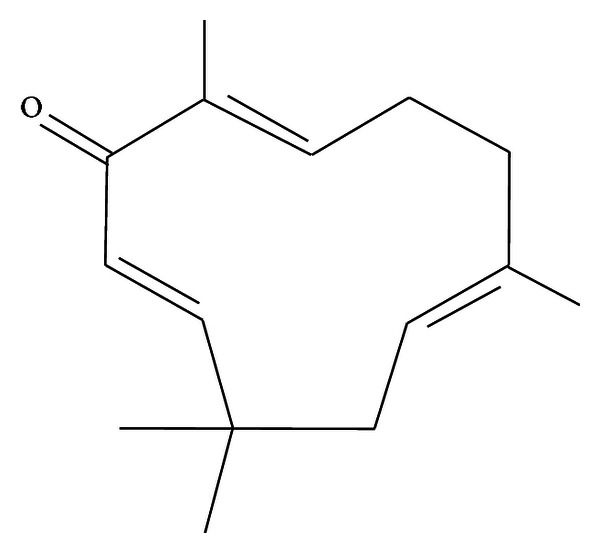
Molecular structure of Zerumbone (2,6,10-humulatrien-1-one).

Zerumbone (ZER) is a crystalline monocyclic sesquiterpene isolated from the rhizomes of *Z. zerumbet*. This bioactive component has a unique structure consisting of a cross-conjugated ketone in an 11-membered ring responsible for its entire biological activities (Figure 1). ZER was previously reported to produce cytotoxicity through apoptosis in various cancers including skin tumor, colon and lung carcinogenesis, hepatocarcinogenesis, leukemic cancer, and cervical intraepithelial neoplasia [10–14].

**Figure 2:**
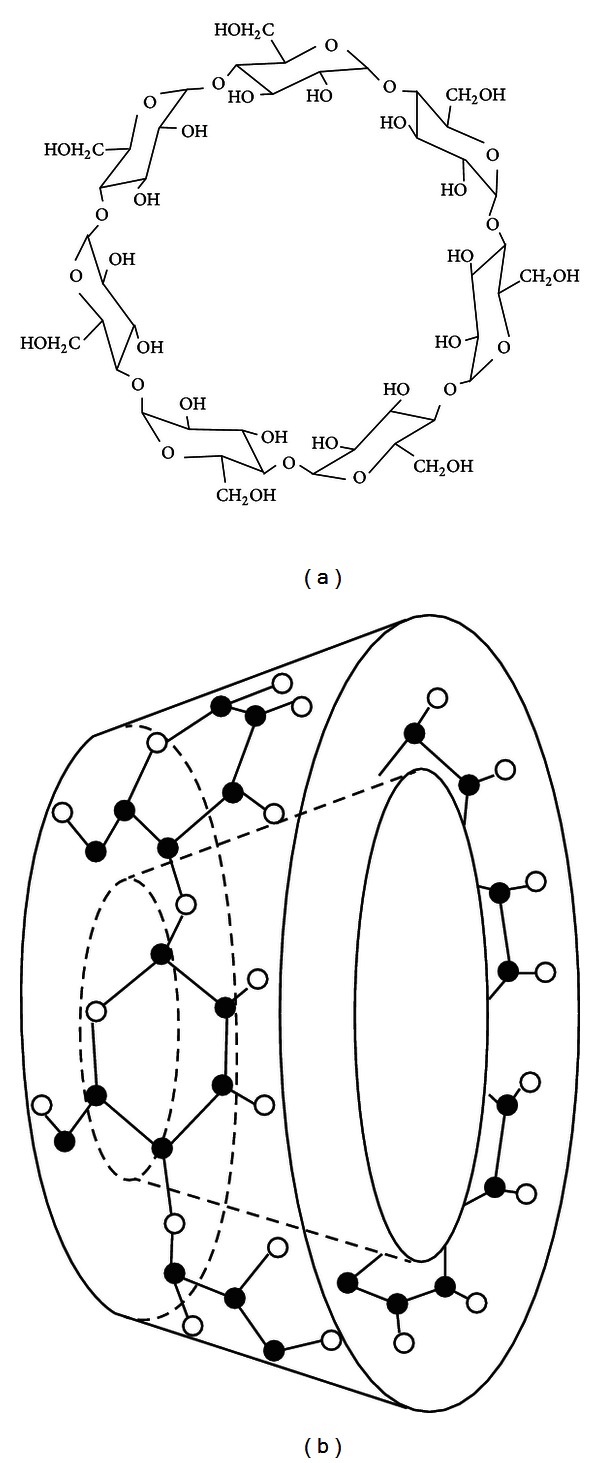
Structure (a) and torus-like shape (b) of *β*-CD molecule.

Hydroxypropyl-*β*-cyclodextrin (HP*β*CD) is a cyclodextrin derivative that is used widely in drug encapsulation due to its inclusion ability as well as its high water solubility (Figure 2). HP*β*CD is well tolerated by the human body both by intravenous and oral administrations. Previously, we investigated the inclusion complex between ZER and HP*β*CD and its physical characterizations. The study provided evidence that ZER penetrates completely into the cavity of HP*β*CD, allowing the solubility of ZER to be enhanced with >30 fold after complexation. The study further showed that HP*β*CD is a suitable encapsular capable of forming a thermodynamically stable complex with ZER for the intended delivery of ZER as an anticancer [15]. Hence, this study further warrants the importance of ZER-encapsulated HP*β*CD as a promising biopharmaceutical drug, which encourages the current study to further investigate the antiproliferative effects of the new ZER-HP*β*CD inclusion complex against liver hepatocellular cells, HepG2, as a potential anticancer. This is the first documented report of ZER-HP*β*CD inclusion complex as an anticancer in HepG2 cells, together with its preliminary investigations of probable molecular mechanism in apoptosis induction.

## 2. Materials and Methods

### 2.1. Cell Lines and Reagents

HepG2, HeLa, MCF-7, MDA-MB-231, CEMss and WRL-68 cells were obtained from American Type Culture Collection (Rockville, MD, USA) and cultured in RPMI1640 (PAA, Germany) media supplemented with 10% fetal bovine serum (FBS) (PAA, Germany) and 1% 100 IU penicillin and 100 *μ*g/mL streptomycin (Sigma, USA). Cultures were maintained in a humidified incubator at 37°C in an atmosphere of 5% CO_2_.

### 2.2. Inclusion Complex of ZER with HP*β*CD

The inclusion complex of ZER with HP*β*CD was obtained from the UPM-MAKNA Cancer Research Laboratory, University Putra Malaysia. Briefly, the thermodynamic parameters (Δ*H, *Δ*S, *Δ*G*) for the formation of the complex were obtained from the van't Hoff equation. ZER complex with HP*β*CD was characterized by differential scanning calorimetry (DSC), X-ray diffractometry (XRD), Fourier transform infrared spectroscopy (FT-IR), Transmission Emission Microscopy (TEM), and molecular modelling using PM6. Calculations show that ZER penetrates completely into the cavity of HP*β*CD [15].

### 2.3. Cytotoxicity Assay

The cytotoxicity profiles of the ZER-HP*β*CD inclusion complex were assessed using 3-[4,5-dimethylthiazol-2-yl]-2,5-diphenyltetrazolium bromide (MTT) microculture tetrazolium viability assay as previously described by Mosmann (1983) with slight modifications [16]. Briefly, cells were seeded in 96-well microplates at a density of 2 × 10^5^ cells mL^−1^. After a 24-hour incubation, the cells were treated at various concentrations of ZER-HP*β*CD inclusion complex (1.563, 3.125, 6.25, 12.5, 25, and 50 *μ*g mL^−1^) for 72 h. After a 68-hour incubation, 20 *μ*L of MTT solution (5 mg mL^−1^) (Amresco, USA) was added into each well and the plate was then incubated for 4 h. Subsequently, the media was removed and the formed formazan crystals were dissolved with 100 *μ*L of DMSO (Sigma, USA). The absorbance was measured at wavelength of 595 nm using a microtiter plate reader (Tecan Sunrise basic, Austria). The percentage of cellular viability was calculated with appropriate controls taken into account. The concentration which inhibits 50% of cellular growth (IC_50_ value) was determined. Three independent experiments performed in triplicates were used for the calculations and statistical analysis.

### 2.4. Morphological Evaluation by Phase Contrast Inverted Microscopy

Treatment was carried out in 25 mL culture flask. HepG2 cells were plated at concentration of 2 × 10^5^ cells mL^−1^ and treated with the ZER-HP*β*CD inclusion complex at 11.43 *μ*g/mL concentration for 24, 48, and 72 h. Morphological appearances of treated cells were compared concurrently with control untreated cells by observing under phase contrast inverted microscopy. Morphological changes that included appearances such as rounding up of cells, plasma membrane blebbing, and cell detachment were observed in treated HepG2 cells.

### 2.5. Propidium Iodide (PI) and Acridine Orange (AO) Double Staining Assay

The AO/PI viability assay is a rapid, highly linear, and functionally correlated assay that has advantages to conventional viability measurement [17]. This analysis examines whether apoptosis may be implicated in mediating cell death in HepG2 cells treated by the ZER-HP*β*CD inclusion complex. After treatment of HepG2 cells with inclusion complex (at 11.43 *μ*g/mL concentration for 24, 48, and 72 h), the cells were harvested and washed with cold PBS twice. The cells were later added with 10 *μ*L of fluorescent dyes, (AO/PI) containing acridine orange (AO, 10 *μ*g/mL), and propidium iodide (PI, 10 *μ*g/mL) at equal volumes of each. Freshly stained cell were observed under confocal microscope (LSM 5 Pascal Zeiss, Germany) within 30 minutes before the fluorescence colour starts to fade.

### 2.6. Assessing Apoptosis Using Annexin V-FITC Assay

Annexin V-FITC Assay was done using the AbD Serotec Annexin V-FITC assay kit (ANNEX100F, USA) in which staining of Annexin V-FITC and propidium iodide was done towards the cells specifically. Fluorochrome FITC-labelled Annexin V is a sensitive protein probe which possesses high affinity towards phosphatidylserine (PS). Briefly, the assay was done according to the manufacturer's instruction. The binding buffer was diluted in 1 : 4 ratios (50 mL binding buffer + 150 mL distilled water). HepG2 cells, at concentration of 2 × 10^5^ cells mL^−1^, were seeded into 25 mL culture flask and treated with the ZER-HP*β*CD inclusion complex at 11.43 *μ*g/mL concentration. After 6, 12, and 24 h of incubation, the cells were harvested and washed with cold PBS twice. Cells were resuspended in prediluted binding buffer and 5 *μ*L Annexin V-FITC was added to 195 *μ*L of the cell suspension, mixed well, and incubated for 10 minutes in the dark at room temperature. The cells were then washed with 190 *μ*L of prediluted binding buffer, followed by the addition of 10 *μ*L of the PI solution, and analysed immediately with flowcytometer (BD FACS Canto II, USA).

### 2.7. Cell Cycle Arrest Analysis

HepG2 cells at concentration of 2 × 10^5^ cells mL^−1^ was seeded into 25 mL culture flask and treated with the ZER-HP*β*CD inclusion complex at 11.43 *μ*g/mL concentration for 24, 48, and 72 h. Untreated cells serve as negative control. The cells were washed with PBS twice to remove any remaining media. To restore cell integrity, fixation of cell population for flowcytometry analysis was performed. Cell pellets were fixed with 90% cold ethanol by mixing 700 *μ*L of 90% cold ethanol and kept for overnight observation at −20°C. The cells were washed using 2 mL PBS twice. Cell pellet was resuspended with 600 *μ*L of PBS + 10 mg/mL RNase + 1 mg/mL propidium iodide (PI). PI has the ability to bind to RNA, and hence, RNAse enzyme was added in order to allow PI to bind directly to DNA. The cells were then incubated between 30 min to 1 h at 37°C. Finally, the cell cycle kinetics was examined using flowcytometer (BD FACS Canto II, USA). The fluorescence intensity of sub-G_1_ cell fraction represents apoptotic cell population.

### 2.8. High Content Screening Assay

This study was conducted using Cellomics Multiparameter Cytotoxicity 3 Kit as described previously [18–20]. Briefly, cells were treated with ZER-HP*β*CD inclusion complex at 11.43 *μ*g mL^−1^ concentration. Untreated cells serve as negative control, whilst paclitaxel (3.68 *μ*g/mL) was used as the positive control. The 96-well microplate was then incubated for 24, 48, and 72 h. In this assay, four important parameters related to apoptosis characteristics were observed simultaneously, which included nuclear morphology changes; changes in cell permeability, mitochondrial membrane potential changes, and cytochrome c release. Plates were analyzed using the ArrayScan HCS system (Cellomics, USA). 

### 2.9. Human Apoptosis Proteome Profiler Array

Detection of the relative levels of apoptosis-related markers was done using Human Apoptosis Antibody Array Kit (RayBio, USA), according to the manufacturer's instruction. Briefly, cells were treated with ZER-HP*β*CD inclusion complex at 11.43 *μ*g/mL concentration. Untreated cells serve as negative control. The human apoptosis array was incubated overnight with 300 *μ*g proteins from each sample. Quantification of the apoptosis array was done using the BiOspectrum AC ChemiHR40 (UVP, Upland, CA, USA) via scanning the membrane, and analysis was done using image analysis software according to the manufacturer's instruction.

### 2.10. Caspase Bioluminescent Assay

Caspase 3/7, caspase 8, and caspase 9 activities were measured in HepG2 cells using a Caspase-Glo assay kit (Promega, USA). HepG2 cells were plated at concentration of 2 × 10^5^ cells mL^−1^ in a white walled 96-well plate and treated with ZER-HP*β*CD inclusion complex at 11.43 *μ*g/mL concentration for 24, 48, and 72 h. Untreated cells serve as negative control. The Caspase-Glo Reagent was mixed well and allowed to equilibrate to room temperature before starting the assay. The 96-well plate containing cells was removed from the incubator and allowed to equilibrate to room temperature as well. Then, 100 *μ*L of Caspase-Glo Reagent was added to each well of the 96-well plate containing 100 *μ*L of blank (vehicle only), negative control cells, or treated cells in culture medium. The contents of wells were gently mixed by using a plate shaker at 300–500 rpm for 30 seconds and incubated at room temperature between 30 min to 3 h. The luminescence of each sample was measured in a luminescence microplate reader (Infinite M200 PRO Tecan, Austria). Concisely, the proluminescent substrate containing the DEVD, LETD, and LEHD (sequence is in a single-letter amino acid code) is cleaved by caspase 3/7, caspase 8, and caspase 9, respectively. After the caspase cleavage, a substrate for luciferase (aminoluciferin) is released which eventually results in the luciferase reaction and the production of luminescent signal analysed in the luminescence microplate reader.

### 2.11. Western Blot Analysis

HepG2 cells were treated with ZER-HP*β*CD inclusion complex for 3, 6, 12, and 24 h. Untreated cells serve as negative control. The total proteins of cells were extracted using cell lysis buffer (50 mM Tris-HCL pH 8.0, 120 mM NaCl, 0.5% NP-40, 1 mM PMSF), and 40 *μ*g of the protein extract was separated by 10% SDS PAGE then transferred into a polyvinylidenedifluoride (PVDF) membrane (Bio-Rad, USA) using semidry transfer unit (Hoefer TE 70X, USA) blocked with 5% nonfat milk in TBS-Tween buffer (0.12 M tris-base, 1.5 M NaCl, 0.1% Tween20) for 1 hour at room temperature. PVDF membrane was then incubated with the appropriate primary antibody overnight at 4°C, then it was incubated with horseradish peroxidase conjugated secondary antibody for 30 minutes at room temperature. The bound secondary antibody was detected with peroxidase-conjugated anti-rabbit antibody (1 : 10000) or anti-mouse antibody (1 : 10000) followed by detection using colorimetric method. The following primary antibodies *β*-actin (1 : 10000), Bcl-2 (1 : 1000), Bax (1 : 1000), and Hsp-70 (1 : 1000) used in this study were purchased from Santa Cruz Biotechnology, Inc., California, USA.

### 2.12. Statistical Analysis

Statistical analysis of all experimental data was performed using Student's *t*-test where *P* < 0.05 was considered statistically significant where results were presented as mean ± SD for at least three analyses for each sample. 

## 3. Results

### 3.1. Antiproliferative Activity

A few types of cell viability were determined by conducting the MTT assay. The IC_50_ values of the ZER-HP*β*CD inclusion complex against five tested cancer cell lines (HepG2, MCF-7, MDA-MB-231, CEMss, and HeLa) including one normal hepatic cell line (WRL-68) are shown in Table 1. The treated HepG2 cells showed a decrement of metabolic activity with an IC_50_ value of 11.43 ± 0.31 *μ*g mL^−1^. The IC_50_ value obtained for pure ZER alone against HepG2 cells was 15.54 ± 0.15 *μ*g mL^−1^, and it shows a similar cytotoxicity effect as exhibited by the ZER-HP*β*CD inclusion complex. Paclitaxel, a commercial anticancer drug, was evaluated as positive control to demonstrate concurrent cytotoxicity with an IC_50_ of 3.68 ± 0.22 *μ*g mL^−1^. According to the American National Cancer Institute, a bioactive compound with an IC_50_ value of ≤30 *μ*g/mL has the potential to be an anticancer agent.

### 3.2. ZER-HP*β*CD Inclusion Complex Showing Morphological Changes on HepG2 Cells That Associate with Apoptosis

**Figure 3:**
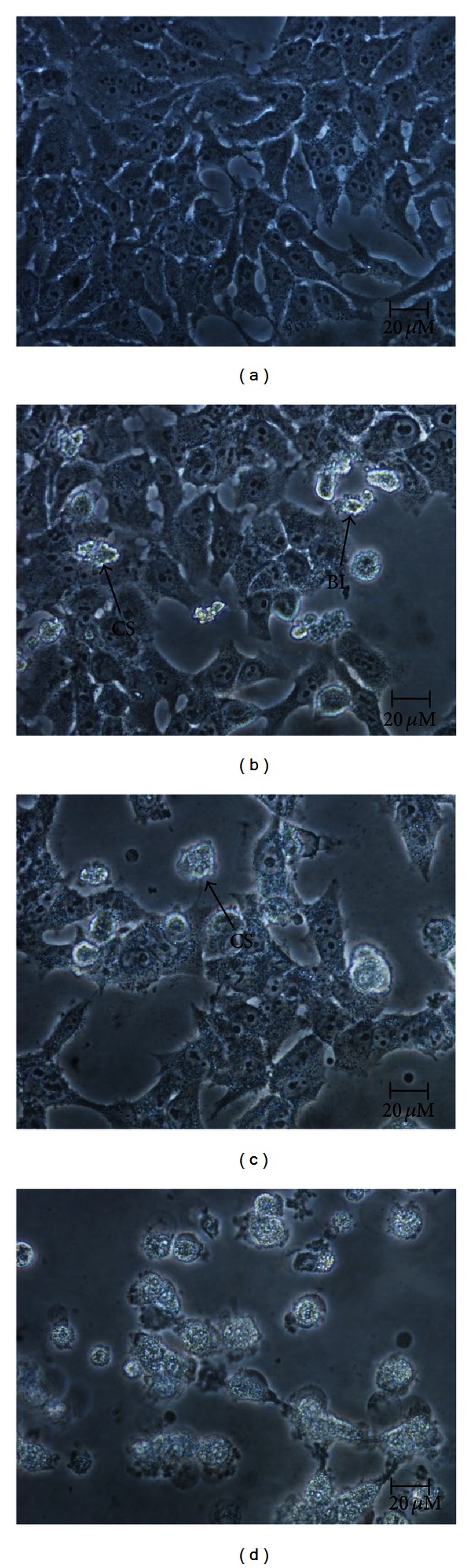
Normal phase contrast inverted micrographs of HepG2 cells treated with 11.43 *μ*g/mL ZER-HP*β*CD inclusion complex. (400x magnification) (a) Control untreated cells; (b) 24 h treatment (most of the cells having normal morphology with few cells showing membrane blebbing); (c) 48 h treatment (detachment of cells, prominent growth inhibition and membrane blebbing showing apoptogenic morphology); (d) 72 h treatment (most of the cells were detached with obvious cell shrinkage). CS: cell shrinkage; BL: cell membrane blebbing.

**Figure 4:**
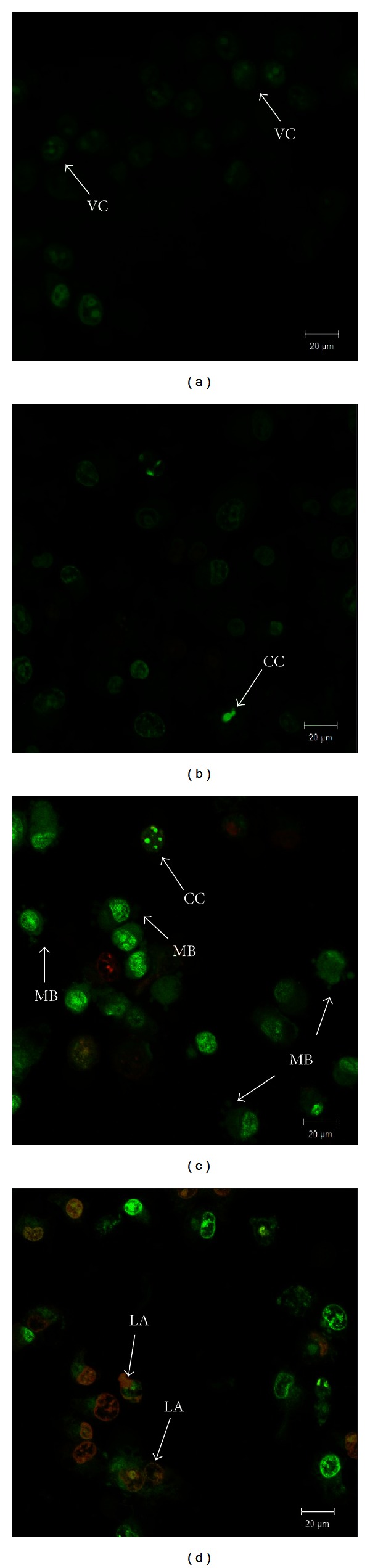
Confocal micrographs of acridine orange and propidium iodide double-stained HepG2 cells treated with 11.43 *μ*g/mL ZER-HP*β*CD inclusion complex. (400x magnification) (a) Untreated cells showed normal structure without prominent apoptosis; (b) early apoptosis features were seen after 24 h representing intercalated acridine orange (bright green) amongst the fragmented DNA; (c) more cells showing membrane blebbing after 48 h; (d) presence of reddish-orange colour at 72 h. VC: viable cells; MB: cell membrane blebbing; CC: chromatin condensation; LA: late apoptosis.

Treatment of ZER-HP*β*CD inclusion complex (11.43 *μ*g/mL) on HepG2 cells showed a cell degenerative in a time-dependent manner while untreated HepG2 control cells were viable and showed normal morphology under normal inverted microscopy (Figure 3). To confirm the apoptosis mechanism of cell death, we examined the nuclear morphological changes using AO/PI double staining. HepG2 cells treated with ZER-HP*β*CD inclusion complex showed cell blebbing and nuclear chromatin condensation of moderate apoptosis after a 24-hour treatment, with subsequent increase at 48 h of treatment followed by the presence of reddish-orange colour after 72 h of treatment due to AO binding towards denatured DNA, thus confirming late stage apoptosis (Figure 4).

### 3.3. ZER-HP*β*CD Inclusion Complex Triggers Early Apoptosis Cell Death in HepG2 Cell Line

Early apoptotic population is indicated by positive Annexin V and negative PI whilst late apoptotic population is indicated by both positive Annexin V and PI. The result obtained is summarized in Table 2. After 6 h of HepG2 cells treatment with ZER-HP*β*CD inclusion complex (11.43 *μ*gmL^−1^), early apoptotic population significantly increased to 9.0%. After 12 h of treatment, early apoptotic population was significantly higher than untreated HepG2 control cells at 8.80% with a decrement of viable cells at 88.80%. After 24 h of treatment, decrement of viable cells continued with 79.30%, and both early and late apoptotic cell populations rose significantly at 15.20% and 5.50%, respectively (*P* < 0.05). These results prove that the treatment of ZER-HP*β*CD inclusion complex on HepG2 cells induced apoptosis, with possible translocation of phosphatidylserine from cytoplasm to the transmembrane of the HepG2 cells.

### 3.4. ZER-HP*β*CD Inclusion Complex Induces Apoptosis with G2/M Phase Cell Cycle Arrest

**Figure 5:**
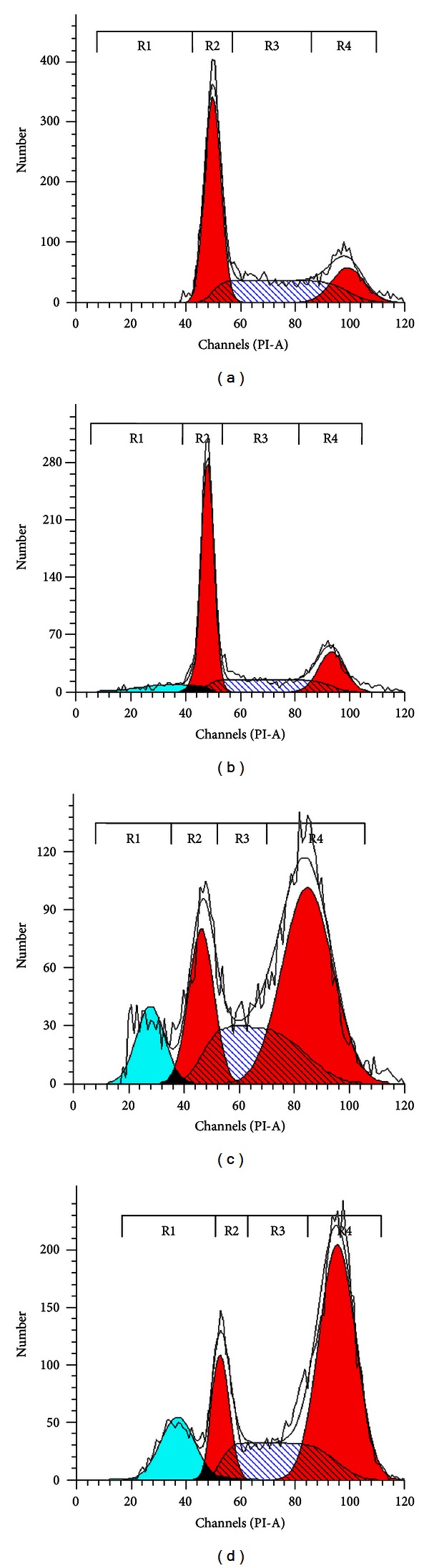
DNA analysis of ZER-HP*β*CD inclusion-complex-treated HepG2. Rapidly proliferating HepG2 cells exposed to 11.43 *μ*g/mL at (b) 24 h, (c) 48 h, (d) 72 h and (a) control were tested for DNA content. Panels are representative of DNA histograms obtained and subsequently analyzed for DNA content; whereby R1, R2, R3, and R5 indicate sub-G0/G1 (apoptosis cells), G0/G1, S, and G2/M phase, respectively.

**Figure 6:**
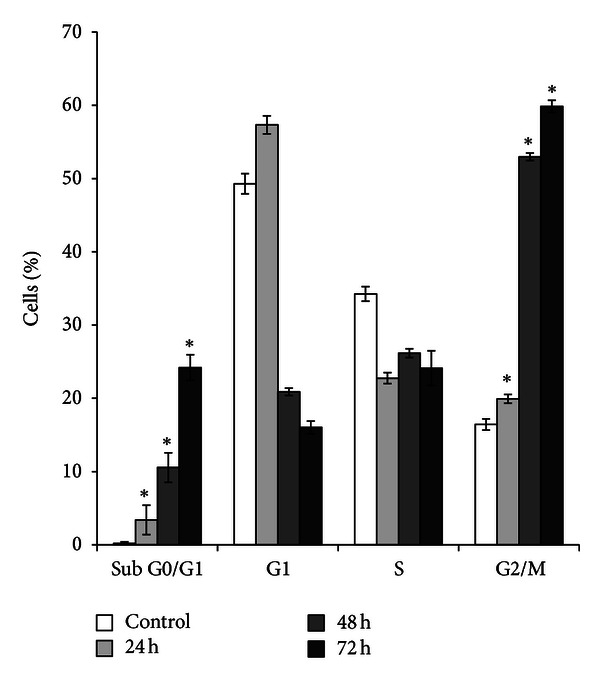
Percentages of cell cycle distribution in sub-G0/G1 (apoptosis cells), G0/G1, S, and G2/M phase of HepG2 cells treated with 11.43 *μ*g/mL of ZER-HP*β*CD inclusion complex for 24, 48, and 72 h. Induction of G2/M arrest in cell cycle progression of HepG2 cells by ZER-HP*β*CD inclusion complex.   *Indicates a significant difference (*P* < 0.05) compared with control.

HepG2 cells treated with ZER-HP*β*CD inclusion complex (11.43 *μ*g/mL) revealed a significant time-dependent increase (*P* < 0.05) in hypodiploid sub-G0/G1 DNA fraction, which corresponds to the presence of apoptotic cells (Figure 6). Sub-G0/G1 fraction in untreated control cells was 0.20%, and this value subsequently increased to 24.20% after 72 h of treatment with ZER-HP*β*CD inclusion complex. The results further indicated that ZER-HP*β*CD inclusion complex treatment caused mitotic block and cell cycle delay in G2/M phase (Figure 5). The proportion of accumulated cells blocked at G2/M phase significantly (*P* < 0.05) increased to 59.84% as opposed to 16.44% for the untreated HepG2 control cells in G2/M phase after a 72-hour treatment (Figure 6).

### 3.5. ZER-HP*β*CD Inclusion Complex Reduces Mitochondrial Membrane Potential and Translocates Cytochrome c in HepG2 Cells

**Figure 7:**
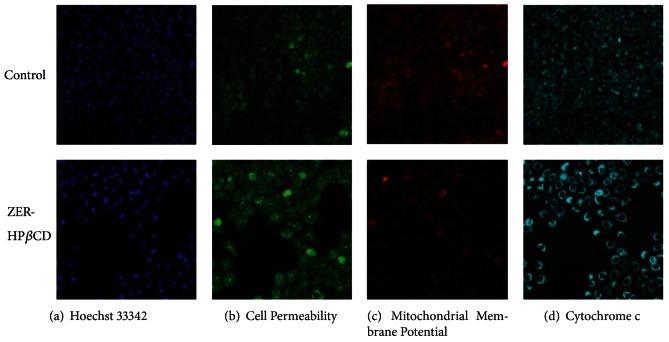
Fluorescent images of HepG2 cells treated with medium alone and 11.43 *μ*g/mL of ZER-HP*β*CD inclusion complex, stained with (a) Hoechst for nuclear, (b) cell permeability dye, (c) mitochondrial membrane potential dye, and (d) cytochrome c. The images from each row were obtained from the same field of the same treatment sample. ZER-HP*β*CD inclusion complex showed marked increases in permeability dye, marked reduction in mitochondrial membrane potential, and marked increases in cytochrome c.

**Figure 8:**
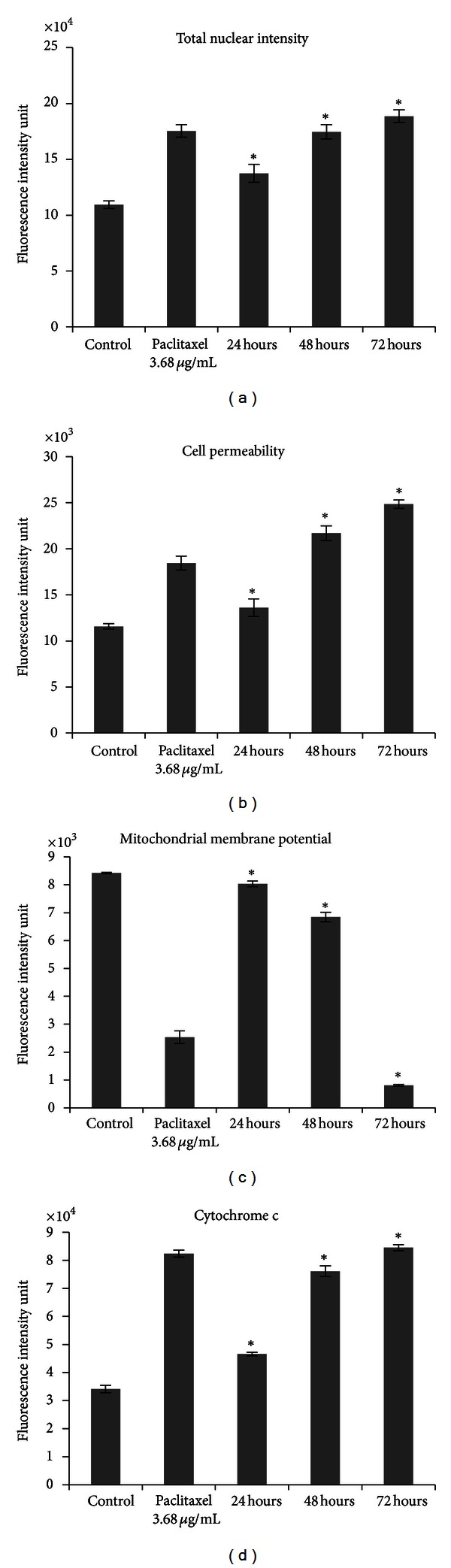
Quantitative analysis of ZER-HP*β*CD inclusion complex mediated apoptosis parameter. Changes in (a) total nuclear intensity, (b) cell permeability, (c) mitochondrial membrane potential, and (d) cytochrome c localization were all measured simultaneously in HepG2 treated cells. Treatment with ZER-HP*β*CD inclusion complex showing statistically significant cell loss (data not shown), increased total nuclear intensity, increased cell permeability, loss of mitochondrial membrane potential, and cytochrome c release from the mitochondria with good *P* values. Each experiment was performed at least two times. *Indicates a significant difference (*P* < 0.05) compared with control.

The nuclear intensity which corresponds to apoptotic chromatin changes (Figures 7(a) and 8(a)) and cell permeability (Figures 7(a) and 8(a)) were both found to be increased significantly (*P* < 0.05) compared to the untreated control cells. Fluorescence intensity in the membrane potential area of HepG2 cells treated with ZER-HP*β*CD inclusion complex (11.43 *μ*g/mL) was shown to be reduced compared to the untreated control cells (Figure 7(c)), reflecting the collapsing of mitochondrial membrane potential with a significant decrease (*P* < 0.05) and a drastic drop after 72 h of treatment (Figure 8(c)). Cytochrome c was released into the cytosol upon treatment, demonstrating possible involvement of the mitochondrial pathway with a significant increase of cytosolic cytochrome c (*P* < 0.05) in a time-independent manner (Figures 7(d) and 8(d)).

### 3.6. ZER-HP*β*CD Inclusion Complex Involves Extrinsic and Intrinsic Apoptosis Pathways

**Figure 9:**
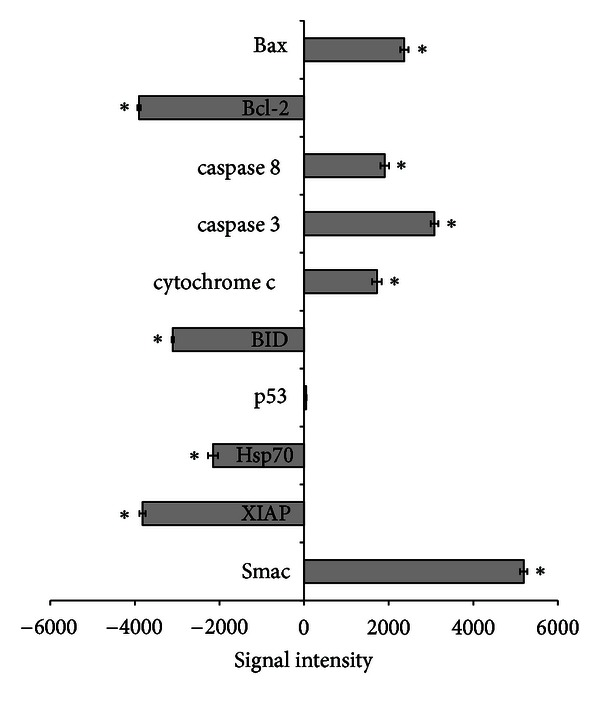
Human apoptosis proteome profiler array analysis in HepG2 cells treated with 11.43 *μ*g/mL of ZER-HP*β*CD inclusion complex for 48 h. Graph shows the difference between treated and control untreated cells. *Indicates a significant difference (*P* < 0.05) compared with control.

The result obtained from our Human Apoptosis Antibody Array analysis showed an upregulation of the proapoptotic Bax protein, which correlated to the downregulation of the antiapoptotic Bcl-2 protein (Figure 9). Caspase 8, caspase 3, and cytochrome c showed significant increase while BID was seen to be depleted significantly after the treatment of HepG2 with ZER-HP*β*CD inclusion complex compared to untreated control cells (*P* < 0.05). The upregulation of caspases 8 and 3 supports the possibility that the ZER-HP*β*CD inclusion complex induces cell death in HepG2 cells via apoptosis with the involvement of both intrinsic and extrinsic pathways. In addition, p53 protein showed no significant change, proving that the apoptosis mechanism was p53 independent (*P* > 0.05). Hsp-70, also an antiapoptotic protein, was found to be significantly decreased (*P* < 0.05). XIAP, an inhibitor of apoptosis protein (IAP) was found to be down-regulated while SMAC that works as the inhibitor to IAP was shown to be up-regulated, providing evidence that both XIAP and SMAC correspond to each other to promote cell death in HepG2 cells. 

**Figure 10:**
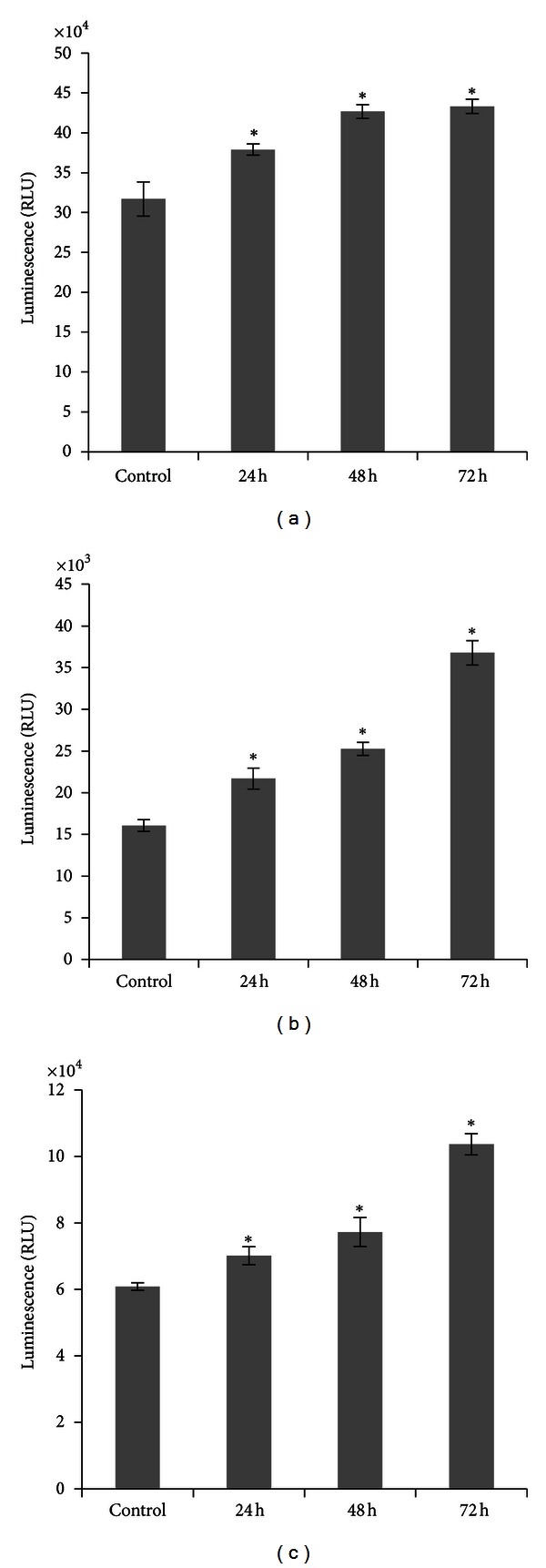
Relative luminescence expression of (a) caspase 3/7, (b) caspase 8, and (c) caspase 9 in the HepG2 cells treated with 11.43 *μ*g/mL of ZER-HP*β*CD inclusion complex for 24, 48, and 72 h. Caspases 3/7, 8, and 9 were significantly increased compared to the control untreated cells. *Indicates a significant difference (*P* < 0.05) compared with control.

The luminescence assay was further used to confirm the involvement of caspases proteins in apoptosis induction. The effector caspase, caspase 3/7, was found to be increased as well as the initiator caspases, caspase 9 and caspase 8 (Figures 10(a), 10(b), and 10(c)). As shown in our results, ZER-HP*β*CD inclusion complex significantly stimulated all three caspases (*P* < 0.05) compared to untreated control cells, further suggesting that both intrinsic (mitochondrial pathway) and extrinsic pathways were involved. These results correspond well with our previous results using the Human Apoptosis Antibody Array analysis. 

**Figure 11:**
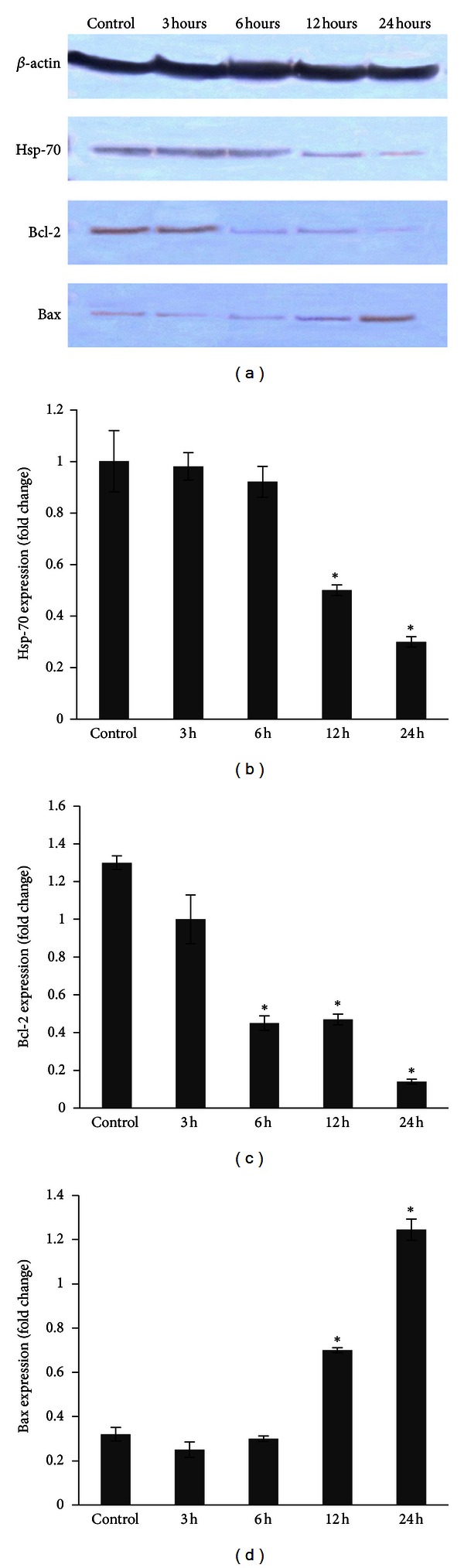
Western blot analysis on effect of ZER-HP*β*CD inclusion complex on the levels of apoptosis regulatory proteins in HepG2 cells. Bax was upregulated while Bcl-2 in contrast downregulated in a time-dependent manner. Hsp-70 was also found to be depleted as time increases. The blot densities are expressed as folds of control. Detection of proteins was done by specific antibodies with *β*-actin as a loading control. Data are mean ± SD (*n* = 3). *P < 0.05 versus control.

The involvement of Hsp-70, Bcl-2, and Bax apoptosis proteins was confirmed using Western blot analysis (Figure 11). Protein Hsp-70 could be seen to decrease gradually after 12 h of treatment without significant changes at 3 h and 6 h of treatment with ZER-HP*β*CD inclusion complex. Similar to Hsp-70, Bcl-2 protein exhibited decreased band intensity in a time-dependent manner, showing obvious fading of the colour after 6 h of HepG2 cells treatment with ZER-HP*β*CD inclusion complex. On the other hand, Bax that acted as proapoptotic protein increased with the increment of treatment time with the highest band intensity after 24 h, providing evidence that treated HepG2 cells initiated the induction of apoptosis cascade mechanism, which finally resulted in cell death. *β*-Actin, used as the loading control, showed equal intensity to all bands, confirming equal protein concentration in all loaded samples.

## 4. Discussion

Due to the growing use of natural-derived substances all around the world, a detailed evaluation of their pharmacological qualities and safety issues is critically needed, since traditional beliefs and remedies cannot stand alone without undergoing detailed scientific studies [21]. Consuming such herbal medicines without proper scientific approval may invite other conflicts which are not favourable to consumers. *Z. zerumbet *is one of the most well-known compounds for its role in traditional medicines as well as in pharmacological activities. ZER is one of the plant bioactive compounds, which can be found mostly in the rhizome of *Z. Zerumbet*, that was previously reported to produce cytotoxicity through apoptosis induction in various cancers including skin tumor, colon, and lung carcinogenesis, hepatocarcinogenesis, leukemic cancer, and cervical intraepithelial neoplasia [10–14].

Despite the apoptotic effects that ZER demonstrates, ZER was reported to be poorly soluble in water and requires organic solvent to solubilize it. Hence, in our previous research, we prepared an inclusion complex of ZER with HP*β*CD and characterized its physicochemical properties. The results provided evidence that ZER fits well inside the nanocavity of HP*β*CD. The study further concluded that the complexation of ZER with HP*β*CD leads to crucial modifications pertaining to the physicochemical properties of ZER which includes its solubility, stability, and bioavailability in blood [15]. Thus, taking into consideration the importance of ZER-HP*β*CD new findings as a potential biopharmaceutical drug, the antiproliferative effects of the inclusion complex was investigated to determine its bioactivity in HepG2 cells. This current study is an initiative to investigate the anticancer activity of the inclusion complex that would eventually lead to preclinical studies of ZER-HP*β*CD inclusion complex as a potential anticancer for future treatment of hepatocarcinoma in humans.

The ZER-HP*β*CD inclusion complex was screened against five different human cancer cell lines: HepG2, MCF-7, HeLa, MDA-MB-231, and CEMss. The results obtained showed that ZER-HP*β*CD inclusion complex exhibits cytotoxic activity on all cancer cell lines screened, with IC_50_ values <30 *μ*g/mL. Pure ZER alone were screened and its activity in HepG2 cells were compared concurrently to that of ZER-HP*β*CD inclusion complex. The determined IC_50_ values confirmed the previous study of pure ZER alone against cell lines (MCF7, HeLa, MDA-MB-231, and CEMss), where it was found to be nearly similar to the IC_50_ values obtained for the ZER-HP*β*CD inclusion complex [15]. These results are consistent with our current study, proving that the cytotoxic activities of ZER-HP*β*CD inclusion complex in these cancer cells may possibly resemble that of pure ZER alone. Interestingly, the ZER-HP*β*CD inclusion complex gave an IC_50_ value of more than 30 *μ*g/mL in the normal hepatic cell line (WRL-68), showing that the cytotoxicity produced by this inclusion complex is selective in cancer cells only. 

The mode of HepG2 cell death was determined based on the characteristics of the cells exerted after treatment. Microscopy analysis has been used as the gold standard for precise detection of cell death, particularly apoptosis, according to the morphological criteria stated by Wyllie et al. (1980) [22] and Yasuhara et al. (2003) [23]. Indications of apoptosis in HepG2 cells treated with ZER-HP*β*CD inclusion complex such as a reduction in the number of cells, detachment of cells, cytoplasmic shrinkage, and membrane blebbing were observed using phase contrast inverted microscopy and confocal microscopy (with AO/PI double staining). The significant increment of apoptotic scores of HepG2 cells treated with ZER-HP*β*CD inclusion complex correlates with a previous study, which reported that HepG2 cells treated with pure ZER lead to a large increase of apoptotic scores at approximately 80% by 48 h and 90% after 72 h [24].

However, morphological studies alone is not sufficient to validate the early and late apoptosis; thus, the finding was further evaluated using the Annexin V assay, which demonstrated that early apoptosis in HepG2 cells was significantly increased in a time-dependent manner after treatment with ZER-HP*β*CD inclusion complex. The critical event during apoptosis is when the changing plasma membrane signals phagocytes to allow them to engulf cells undergoing apoptosis before rupturing [25]. Modification of the apoptotic cell surface included the exposure of phosphatidylserine (PS), which normally dominates surface membrane facing the cytosol [26]. PS externalization occurs in early apoptosis event before the cell undergoes nuclear changes, regardless of the initiator apoptosis catalyst [27]. Hence, early phases of apoptosis (prior to the loss of cell membrane integrity) were detected using the fluorescein isothiocyanate (FITC) conjugated Annexin V (protein with high affinity for PS) binding assay of PS, allowing measurement and therefore scoring of individual apoptotic cells [25]. Our results in this current study are parallel with the significant increase of apoptosis in HepG2 cells at 6, 12, and 24 h treatments, which proved that the ZER-HP*β*CD inclusion complex is able to trigger early apoptosis with the exposure of PS at the external surface of treated HepG2 cells.

To further elucidate the probable mechanisms of apoptosis induced by ZER-HP*β*CD inclusion complex in HepG2 cells, cell cycle analysis was performed and evaluated. The observed results showed a hypodiploid sub-G0/G1 DNA accumulation, which concluded that the ZER-HP*β*CD inclusion complex is able to induce apoptosis in HepG2 cells in a time-dependent manner and simultaneously induce cell cycle arrest at G2/M. This mitotic blockage has been observed as a result of treating HepG2 cells with the accumulation of ZER-HP*β*CD inclusion complex at G2/M. It has been reported that the G2/M arrest triggers possible phosphorylation of apoptosis-associated proteins in the mitotic phase of cell cycle, which further explains the involvement of G2/M arrest to be associated with apoptosis [28]. This result was confirmed by previous studies done on pure ZER alone, which was found to inhibit interleukin-6 and induces apoptosis and cell cycle arrest at G2/M phase in ovarian (Caov-3) and cervical (HeLa) cancer cells [29]. It was also previously reported elsewhere that ZER concurrently showed apoptogenic effects to induce G2/M cell cycle arrest in promyelocytic leukemia NB4 cells and colon adenocarcinoma HT-29 cell line [30, 31].

A molecular study was conducted to clarify probable mechanisms of apoptosis induced by ZER-HP*β*CD inclusion complex using high-content screening assay, proteome profiler array, caspase luminescence assay, and Western blot analysis. The study confirmed that ZER-HP*β*CD inclusion complex stimulates apoptosis signal, causing a decrement of membrane permeability in HepG2 cells followed by mitochondrial transmembrane potential (ΔΨm) changes. The structure and dynamics of the altered cell membrane in HepG2 cells are due to this increment of susceptibility to hydrolysis; thus, increasing cell permeability and finally events leading to apoptosis [32]. Any stimulus causing apoptosis affects HepG2 cells in such a way that will cause permeability transition pores to open to the inner mitochondrial membrane, subsequently causing increased permeability of the inner mitochondrial membrane capable of soluting molecular mass of less than about 1500 Da, thus resulting in the depletion of ΔΨm, and in turn, the release of proapoptotic proteins and the arrest of the bioenergetic function of the organelle [33–35].

The present study further demonstrated that the exposure of HepG2 cells to ZER-HP*β*CD inclusion complex showed an upregulation of proapoptotic protein, Bax, and a downregulation of antiapoptotic, Bcl-2 protein. Bax and other related proapoptotic proteins activities were affected by the formation of Bcl-2 antiapoptotic protein. Bax will be translocated to the mitochondria and other membrane sites, which activates the transformation of mitochondrial function. This mitochondrial transformation will cause loss of transmembrane potential and release of cytochrome c to the cytosol, resulting in apoptotic cell death [36]. Overexpression of Bcl-2 will prevent the release of cytochrome c, causing HepG2 cells to be resistant to apoptotic induction [37]. Contrary to this, ZER-HP*β*CD inclusion complex was able to suppress Bcl-2 expression, thus allowing Bax to form a homodimer with another Bax instead of forming a heterodimer with Bcl-2. These upregulation and downregulation of Bax and Bcl-2 ratio reaffirm a previous study which reported that pure ZER alone induced apoptosis in HepG2 cells via modulation of Bax/Bcl-2 ratio [36].

Results of the caspase luminescence assay and apoptosis proteome profiler array further demonstrated that ZER-HP*β*CD inclusion complex treatment induces the upregulation of caspase 8, caspase 9, caspase 3, and cytochrome c, and degradation of BID in treated HepG2 cells. The upregulation of caspase 8 was due to the proteolysis of procaspase 8, forming activated caspase 8 and therefore allowing the amplification of caspase signal for apoptosis [38]. In a previous study elsewhere, commercial drugs such as daunorubicin, doxorubicin, etoposide, and mitomycin C induced apoptosis in Jurkat leukemic T cells involving the activation of caspase 8 [39]. Paclitaxel was also reported to induce apoptosis involving caspase 8 activation in colon cancer cell (HT-29-D4) [40]. Activated caspase 8 will cleave BID into a proapoptotic fragment called truncated BID (tBID), which will later translocate to the mitochondrial membrane with a subsequent release of cytochrome c into the cytosol with the help of Bax [41]. Cleavage of BID is mediated by caspase 8, which is known to connect the extrinsic and mitochondrial pathways in apoptosis cell death [42]. Cytochrome c release will induce the oligomerization of cytochrome c/Apaf-1/caspase 9 complex that activates caspase 9 and finally leads to the cleavage of downstream effector caspase 3 and 7 [43].

The protein expression of p53 did not exhibit any significant changes in HepG2 cells treated with ZER-HP*β*CD inclusion complex, providing evidence for the first time that induced apoptosis upon treatment of HepG2 cells with ZER-HP*β*CD inclusion complex is p53 independent. Although p53 is known as the “guardian of the genome” since it prevents proliferation of damage cells, several chemotherapy drugs such as paclitaxel, tamoxifen, and vinkristin were identified as able to induce apoptosis without the involvement of p53 [44–46]. This is probably due to the expression of Bax protein, which after achieving certain level is able to induce apoptosis without the presence of p53 [44]. Our current finding is crucial as most cancer tumours including those of the breasts, lung, colon, bladder, brain, bone, hematopoietic, and muscle tissues are initiated due to the abnormalities of or mutated p53 gene [47]. This would allow the ZER-HP*β*CD, a significant anticancer, complex to be able to trigger the induction of apoptosis in most human cancers that are p53 dependent.

Heat shock protein (Hsp) 70 will interfere with the apoptotic process of cell death as its role is to mediate cellular protection by preventing cytochrome c/dATP-mediated caspase activation but allowing the formation of Apaf-1 oligomers to be accessible. Hsp-70 binds to Apaf-1 but not to procaspase 9, therefore preventing the recruitment of caspases to the apoptosome complex. This directly blocks the assembly of functional apoptosome and prevents the cell from undergoing apoptosis [48]. In this current study, Hsp-70 was found to be suppressed in HepG2 treated with ZER-HP*β*CD inclusion complex, resulting in the Apaf-1 functional assembly blockage to be prevented. Protein Hsp-70 was also found to be decreased gradually only after 12 h of treatment without any significant changes at 3 h and 6 h of treatment. In accordance with this, it may be suggested that the host stress response of HepG2 cells may be activated at 3 and 6 h of treatment but with Hsp-70 exhibiting no significant increase during this period due to the treatment of ZER-HP*β*CD inclusion complex. It was reported previously that, in normal unstressed cells, Hsp-70 is expressed only at a very low level and nearly undetectable while in most human tumors, it is expressed at a very high level while adjusting to the unfavourable environmental conditions [49–51]. With the ability to suppress Hsp-70 in HepG2 cells, ZER-HP*β*CD inclusion complex will be able to stop the growth of HepG2 cells which finally leads to apoptosis of the cells.

Procaspase activation and caspase activity need to be controlled in order to prevent overexpression of such proteins. In addition, the inhibition of apoptosis protein (IAPs), which acts as the modulator to directly control caspase activation, inhibits caspase 3, caspase 7, and caspase 9 activities. Among all IAPs identified, X-linked inhibitor of apoptosis protein (XIAP) is the most crucial in suppressing procaspase activation [52]. Hence, in order to induce cell death via apoptosis, protein suppression by XIAP must be crucially blocked. In this regard, the second mitochondria-derived activator of caspases (Smac)/DIABLO resumes the responsibility to suppress XIAP activity. As proapoptotic of Bcl-2 family induces the mitochondrial transmembrane potential (ΔΨm) changes, Smac/DIABLO protein is released from the mitochondria [53]. ZER-HP*β*CD inclusion complex has shown the ability to induce XIAP inhibition through Smac protein activation in HepG2 cells and therefore allowing the cascade caspases to be activated downstream which in turn triggered apoptosis induction.

## 5. Conclusion

Collectively, our current findings showed that the highly soluble ZER-HP*β*CD inclusion complex possesses anticancer properties towards HepG2 cell line and the death receptor pathway may be involved in the induction of apoptosis. Our current finding can be extrapolated to postulate that caspase 8's activation is indirectly involved as an interconnection between the extrinsic and intrinsic pathways. This ZER-HP*β*CD inclusion complex not only demonstrated antiproliferative effects towards HepG2 cells, but its high solubility in water provided the advantage to pursue the complex as a therapeutic drug candidate in humans.

Future *in vivo* studies are recommended prior to the use of the ZER-HP*β*CD inclusion complex as an anticancer on hepatocarcinoma patients, and detailed toxicity studies of this complex are vital before pursuing human clinical trials.

## Figures and Tables

**Table 1:** Cytotoxicity of ZER-HP*β*CD inclusion complex on different cancer and normal cells *in vitro*.

Cell line	Origin of cells	IC_50_ (*μ*g/mL)
HepG2	Liver hepatocellular cells	11.43 ± 0.31
MCF-7	Oestrogen receptor-positive breast adenocarcinoma cells	15.32 ± 0.61
MDA-MB-231	Oestrogen receptor-negative breast adenocarcinoma cells	17.20 ± 0.17
CEMss	T-acute lymphoblastic leukemia	9.13 ± 0.38
HeLa	Cervical cancer cells	14.47 ± 0.26
WRL-68	Normal hepatic cell line	>30

IC_50_ values were obtained from MTT assay. Data are reported as means ± SD for measurements in triplicate.

**Table 2:** Flow cytometric analysis of Annexin V in HepG2 cells which were treated with 11.43 *μ*g/mL ZER-HP*β*CD inclusion complex for 6, 12, and 24 hours.

Cell condition	Percentage of cells (%)			
	Control	6 h	12 h	24 h
Viable	93.90 ± 1.20	89.70 ± 1.50	88.80 ± 3.20	79.30 ± 5.80
Early apoptosis	4.20 ± 0.27	9.00 ± 0.31*	8.80 ± 0.44*	15.20 ± 0.40*
Late apoptosis	1.90 ± 0.12	1.30 ± 0.53	2.30 ± 0.35	5.50 ± 0.27*

Data are shown as mean ± SD (*n* = 3); *Indicates a significant difference compared with control (*P* < 0.05).

## Abbreviations

AO: Acridine orange
Apaf-1: Apoptotic protease-activating factor-1
Bax: Bcl-2 associated X protein
Bcl-2: B cell lymphoma 2
BID: BH3 interacting domain death agonist
DMSO: Dimethyl sulfoxide
FITC: Fluorescein isothiocyanate
HP*β*CD: Hydroxypropyl-*β*-cyclodextrin
Hsp-70: Heat shock protein 70
IC_50_: Half maximal inhibitory concentration
MMP: Mitochondrial membrane potential
MTT: 3-(4,5-Dimethylthiazol-2-yl)-2,5-diphenyltetrazolium bromide
PI: Propidium iodide
PS: Phosphatidylserine
SMAC: Second mitochondria-derived activator of caspases
XIAP: X-linked inhibitor of apoptosis protein
ZER: Zerumbone
ΔΨm: Mitochondrial transmembrane potential.
